# Integrative analysis of transcriptome and proteome revealed nectary and nectar traits in the plant-pollinator interaction of *Nitraria tangutorum* Bobrov

**DOI:** 10.1186/s12870-021-03002-9

**Published:** 2021-05-22

**Authors:** Tingting Chen, Yanwei Zhou, Jingbo Zhang, Ye Peng, Xiuyan Yang, Zhaodong Hao, Ye Lu, Weihuang Wu, Tielong Cheng, Jisen Shi, Jinhui Chen

**Affiliations:** 1 Key Laboratory of Forest Genetics and Biotechnology of Ministry of Education of China, Co-Innovation Center for Sustainable Forestry in Southern China, Nanjing Forestry University, 159 Longpan Rd, Xuanwu Nanjing, 210037 China; 2 Experimental Center of Desert Forestry, Chinese Academy of Forestry, Dengkou, Inner Mongolia China; 3 College of Biology and the Environment, Nanjing Forestry University, Nanjing, 210037 China; 4 Research center of saline and alkali land of National Forestry and Grassland Administration, Chinese Academy of Forestry, Beijing, 100091 China

**Keywords:** Ecological plant, Entomophilous, Nectary development, Antibacterial protein

## Abstract

**Background:**

*Nitraria tangutorum* is an important desert shrub that shows resistance to drought, salt and wind erosion stresses. It is a central ecological species in its area. Here, we have studied how *N. tangutorum* has adapted to achieve a successful reproduction strategy.

**Results:**

We found that *N. tangutorum* is mainly pollinated by insects of the Hymenoptera, Diptera and Coleoptera orders. *Nitraria tangutorum* has very small flowers, with the nectary composed of secretive epidermal cells from which nectar is secreted, located within the inner petals. In addition, analyzing the transcriptome of four successive flower developmental stages revealed that mainly differentially expressed genes associated with flower and nectary development, nectar biosynthesis and secretion, flavonoid biosynthesis, plant hormone signal transduction and plant-pathogen interaction show dynamic expression. From the nectar, we could identify seven important proteins, of which the L-ascorbate oxidase protein was first found in plant nectar. Based on the physiological functions of these proteins, we predict that floral nectar proteins of *N. tangutorum* play an important role in defending against microbial infestation and scavenging active oxygen.

**Conclusions:**

This study revealed that *N. tangutorum* is an insect-pollinated plant and its nectary is composed of secretive epidermal cells that specialized into secretive trichomes. We identified a large number of differentially expressed genes controlling flower and nectary development, nectar biosynthesis and secretion, flavonoid biosynthesis, plant hormone signal transduction and plant-pathogen interaction. We suggest that proteins present in *N. tangutorum* nectar may have both an antibacterial and oxygen scavenging effect. These results provide a scientific basis for exploring how the reproductive system of *N. tangutorum* and other arid-desert plants functions.

**Supplementary Information:**

The online version contains supplementary material available at 10.1186/s12870-021-03002-9.

## Background

Pollination success rate is key to plant reproduction and therefore is one of the main focus of plant reproductive ecology and evolutionary biology. The movement of pollen largely determines the gene exchange rates between plants and provides a basis for the study of species evolution [1]. Usually, plants can use insects as carriers for fertilization to achieve more accurate and efficient transmission of genetic information. Especially for smaller floral organs, which makes artificial pollination very inconvenient. Therefore, auxiliary pollination by insects is one of the main focuses. For attracting insects, flower traits, nectary morphology and nectar characteristics are the most important factors, and they play a critical role in the relationship between plants and pollinators [2].

The nectary is a secretory organ possessed by virtually all existing flowering plants [3, 4]. In different plant species, the nectary varies in shape and size, but the internal structure is often similar, usually composed of a layer of secretory epidermal cells and several layers of nectar secreting tissue cells [5]. The position and structure of the nectary can be tied to different floral structures, further influencing pollinator behavior [6]. By way of example, floral nectar spurs can limit pollinator type, thereby promoting reproductive isolation; tubular flowers for example tend to be pollinated by hummingbirds [7]. Previous studies have found that a number of developmental genes are expressed in the nectary and required for its development, such as *CRABS CLAW* (*CRC*), *LEAFY* (*LFY*), *UNUSUAL FLORAL ORGANS* (*UFO*), *BLADE-ON-PETIOLE1* (*BOP1*), *BLADE-ON-PETIOLE2* (*BOP2*), *APETALA2* (*AP2*), *AGAMOUS* (*AG*), *SHATTERPROOF* (*SHP*)*1* and *2*, *AGL1/SHP1*, *MYB24*, *MYB57* and *PIN6* [8–15].

It is widely known that nectar is not only a direct source of honey, but also the basis for the survival of many insects. When insects feed on nectar, their bodies will carry pollen. Therefore, the nectar’s characteristics will also directly affect the types of pollinators and pollination efficiency. Nectar is a mixture of sugars, proteins, amino acids, polyphenols, lipids and other substances, with components varying between different species [16]. When nectar is secreted, it accumulates at the base of the flower or at a particular distance. The exposed nectar tends to attract beetles, flies, short-snouted bees and moths. However, the nectar hidden deep within the corolla mostly attracts long-snouted butterflies and moths [17]. The volume of nectar also attracts different pollinators, for example, bumblebees and bats prefer high-volume nectar, while the hoverfly and small solitary bees prefer low-volume nectar [18]. Similarly, the consistency of nectar will also affect the pollinator type. Previous studies have found several genes to be involved in regulating nectar synthesis and secretion, such as *CELL WALL INVERTASE4* (*CWINV4*), *MYB21*, *MYB305*, *NEC1*, *NEC3*, *JMTNTR1*, *SWEET9 and CYP86B1* [19, 20]. Moreover, some nectar proteins have antibacterial, oxygen scavenging or energy metabolism functions, mainly include Nectarin I, II, III, IV, V, class II and III chitinases (Chi2, Chi3), superoxide dismutase (SOD), monodehydroascorbate reductase (MDAR), peroxidase (POD), rubber elongation factor protein (REF), non-specific lipid-transfer protein (nsLTPs) and fructose-1,6-bisphosphatase (FBP2) [21–24].

*Nitraria tangutorum* survives and grows in the arid-desert areas of northwestern China since a long time and has developed a strong tolerance to drought, salt and wind erosion stresses [25]. Its leaves contain a variety of mineral elements and volatile oils, and are richly nutritious and well palatable, making it a suitable forage plant in an arid area [26]. Furthermore, the fruits of *N. tangutorum* help to lower blood pressure and blood sugar [27, 28]. Therefore, *N. tangutorum* has interesting ecological and economic prospects. Furthermore, its reproduction process has developed an adaptation to the surrounding environmental conditions, it can produce seeds for species maintenance. Therefore, understanding its reproduction process is of crucial importance. For these reasons, here we comprehensively study its ecological characteristics of reproduction through various surveys and experiments, including studies on pollinator species and their behavior, morphology and development of the nectary, the genes associated with nectary development and nectar secretion, as well as identification of nectar proteins. This study broadens the existing knowledge on the relationships between flowers and their pollinating agents and contains new data on the molecular and metabolic mechanisms underlying the synthesis of nectar and how it assists in defense against microbes. Therefore, it is critical from an ecological point of view, benefiting further breeding studies on arid-desert plants.

## Results

### The flower development and behavioral patterns of floral visitors in *Nitraria tangutorum*

**Fig. 1:**
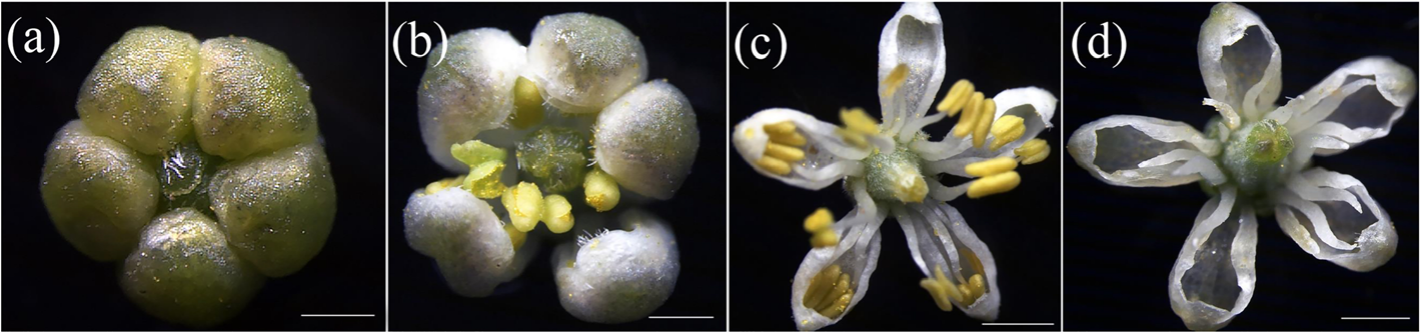
Floral morphology of successive *Nitraria tangutorum* flower developmental stages. **a** S1: stage 1, the petals form a tightly enclosing structure, **b** S2: stage 2, the petals begin to expand and split, **c** S3: stage 3, the androecium surrounds the gynoecium, **d** S4: stage 4, pollination was successful and small fruit has appeared. Scale bar =1 mm (**a**-**b**) or 2 mm (**c**-**d**)

*Nitraria tangutorum* flowers are small in stature and bisexual with the gynoecium surrounded by the androecium. We observed that the flowering period is from May to June and that the full blooming phase lasts approximately 3–5 days. To characterize flower development, we observed its flowers at four morphologically distinct stages. During the squaring period, the flower petals (with 3 mm in height and 2 mm in diameter) were densely arranged (Fig. 1a, stage 1). Then, the petals began to expand and split during the early flowering period, and the yellow stamina gradually emerged. At this stage, the height and diameter of the flower were both 3 mm (Fig. 1b, stage 2). During the full blooming period, each flower clearly showed five radially arranged small white petals, their diameter ~ 5 mm and their height constant. (Fig. 1c, stage 3). At the end of the blooming period, the petals and stamens gradually withered and fell off, leaving the ovary exposed, which will later develop into fruit (Fig. 1d, stage 4).

**Fig. 2:**
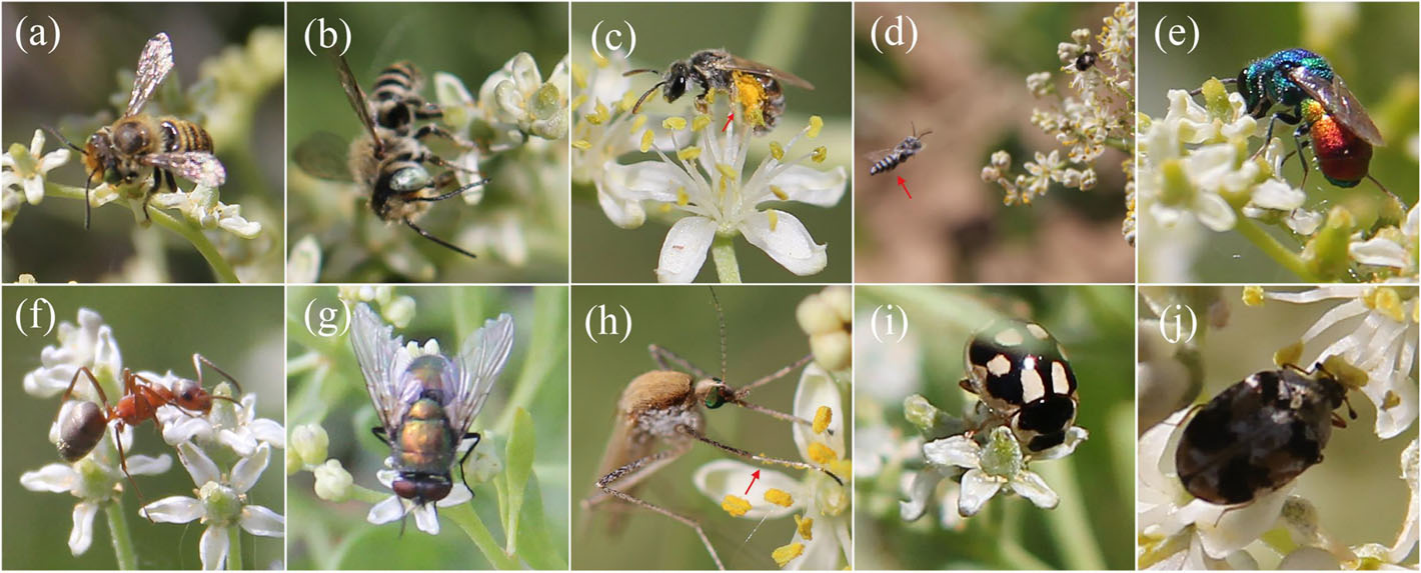
Pollinator’s foraging behaviors on *Nitraria tangutorum* flowers. **a**-**j** Different orders of insect act as pollinators on *Nitraria tangutorum* flowers. **a**-**c** *Apidae* (Hymenoptera), note the abdomen covered in pollen in (**c**) (arrow), **d** *Megachilidae* (Hymenoptera), **e** *Pteromalidae* (Hymenoptera), **f** *Formicidae* (Hymenoptera), **g** *Muscidae* (Diptera), **h** *Culicidae* (Diptera), note its legs covered in pollen (arrow), **i** *Coccinellidae* (Coleoptera), **j** *Chrysomelidae* (Coleoptera)

All insects foraged for nectar or pollen during the full blooming period. We observed while the insects collected nectar or pollen, they pollinated flowers. When visiting flowers, they grasped petals by using their feet to fix the body, generally staying on a single flower for 10–30s, then flying to another flower. Their visits took place between 9:00–17:00. According to our observations and records on the floral visitors, they were mainly Hymenoptera, Diptera and Coleoptera. Hymenoptera visitors include *Apidae* (Figs. 2a-c), *Megachilidae* (Fig. 2d), *Pteromalidae* (Fig. 2e) and *Formicidae* (Fig. 2f). Diptera visitors include *Muscidae* (Fig. 2g) and *Culicidae* (Fig. 2h). Coleoptera visitors include *Coccinellidae* (Fig. 2i) and *Chrysomelidae* (Fig. 2j). They were able to collect nectar from the nectary by using their mouthparts. The dense villi on the bee’s body surface could adhere to pollen, thus completing the pollination. We also found that bees carried pollen blocks on their abdomen (Fig. 2c), while mosquitoes carried pollen on their legs (Fig. 2h). Based on our observations, we found that excluding ants that may act as nectar thieves, other floral visitors were found to be effective pollinators that could perform pollination while they were collecting pollen or sucking nectar.

### Characteristics of the *Nitraria tangutorum* nectary structure

**Fig. 3:**
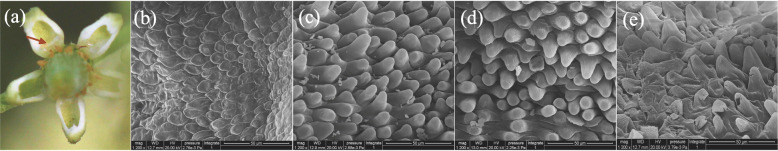
*Nitraria tangutorum* floral nectary morphology and development. **a** Nectar (red arrow), **b** S1: secretory epidermal cells are at the beginning of development, **c** S2: secretory epidermal cells are prominent and specialized to a papillae-like structure, **d** S3: secretory epidermal cells showing papillae with the striate cuticle, **e** S4: secretory epidermal cells shrink and show the striate cuticle

When fixing the flower samples for nectary structure observation, we found clear liquid was distributed at the base of the petals (Fig. 3a). At stage 1, the secretory epidermal cells were not obvious but had regular surface. They were elliptic but not yet protruding (Fig. 3b). Afterwards, at stage 2, the secretory epidermal cells clearly started to differentiate from their surrounding cells, gradually protruding outward. The tips of the cells were brighter, possibly due to the nectar that was about to secrete (Fig. 3c). At stage 3, the secretory epidermal cells showed a large number of short papillae-like protuberances. Also, secretory epidermal cells showed a striate cuticle (Fig. 3d). At stage 4, the number and length of the secretive trichomes reached its maximum. The secretory epidermal cells gradually began to shrink and showed their obvious striate cuticle, finally falling off together with the petals (Fig. 3e).

### Global transcriptomic changes and comparative analysis of four successive flower developmental stages

Based on our previous phenotypic observations on the *N. tangutorum* nectary and its pollinators, we asked whether some key genes might play a significant role during flower development. We performed RNA-seq on samples from the four successive stages of flower development, applying three biological replicates at each stage. In total, we sequenced 12 samples on the BGISEQ-500 platform and generated approximately 6.22Gb of sequencing per sample. On average 72.97% reads were mapped (Table S1). We then determined the Pearson Correlation between all samples (Fig. S1a), finding that replicate samples correlated strongly and confirming the consistency of our RNA-seq data. Gene expression levels built on Fragments Per Kilobase of exon per Million reads mapped (FPKM) values and gene annotation were shown in Dataset 1. Gene expression distribution for each sample was shown in Fig. S1b. We identified preliminary differentially expressed genes (DEGs) by comparing the different developmental stages (Fig. S1c).

**Fig. 4:**
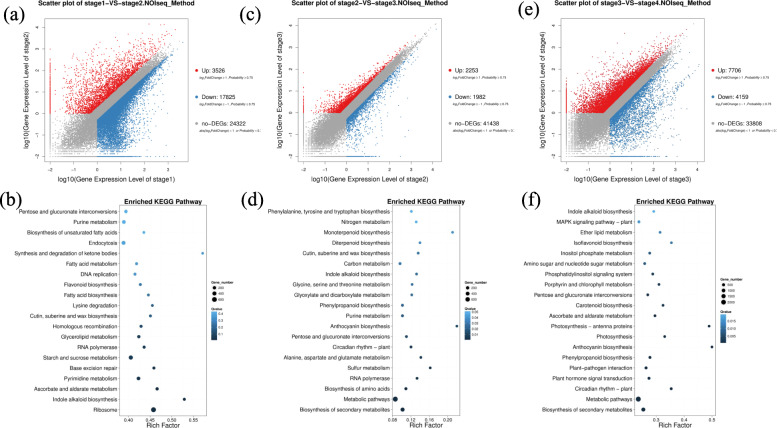
Functional enrichment of differentially expressed genes. **a**, **c**, **e** Scatterplots showing the number of DEGs identified and their expression levels between stages 1 and 2 (**a**), 2 and 3 (**c**), 3 and 4 (**e**). **b**, **d**, **f** KEGG functional enrichment analysis of DEGs identified between stages 1 and 2 (**b**), 2 and 3 (**d**), 3 and 4 (**f**). Gene differential expression analysis was performed using NOIseq (https://bioinfo.cipf.es/noiseq/doku.php?id=downloads). The significance threshold was set at |Log_2_FoldChange| ≥ 1 and Probability ≥0.75. Functional interpretation was further completed by KEGG (http://www.genome.jp/kegg/pathway.html)

To analyze the functionality of genes differentially expressed between successive developmental stages, we made three separate stage comparisons. First, between stages 1 and 2 (transition period:1–2 days), 21,351 DEGs were identified, with 3526 up-regulated genes and 17,825 down-regulated genes, the largest number of DEGs out of all three comparison, showing the high expression dynamics of the first stage transition (Fig. 4a). KEGG enrichment revealed that the main functions of these genes were ribosome biogenesis, indole alkaloid biosynthesis, ascorbate and aldarate metabolism, starch and sucrose metabolism and flavonoid biosynthesis (Fig. 4b).

Second, between stages 2 and 3 (transition period: one half day), 4235 DEGs were identified, with 2253 up-regulated genes and 1982 down-regulated genes, the lowest number of DEGs out of three stage transitions (Fig. 4c). There was no obvious difference between the number of up-regulated and down-regulated genes. KEGG enrichment analysis of this second transition mainly revealed DEGs to be classified to metabolic pathways, biosynthesis of secondary metabolites, biosynthesis of amino acids, circadian rhythm, pentose and glucuronate interconversions and anthocyanin biosynthesis (Fig. 4d).

Finally, between stages 3 and 4 (transition period: 3–5 days), we identified 11,865 DEGs, with 7706 up-regulated genes and 4159 down-regulated genes (Fig. 4e). A large number of genes showed upregulation at stage 4. The more significant pathways were biosynthesis of secondary metabolites, metabolic pathways, circadian rhythm, plant hormone signal transduction, plant-pathogen interaction, phenylpropanoid biosynthesis and anthocyanin biosynthesis. In addition, we identified some photosynthesis-related pathways, such as photosynthesis-antenna proteins, photosynthesis, porphyrin and chlorophyll metabolism (Fig. 4f). During this period, the petals gradually fall off. The fruits develop through accumulating carbohydrates via photosynthesis and resist pathogens to ensure normal development.

### Time-course RNA-seq analysis during flower development

**Fig. 5:**
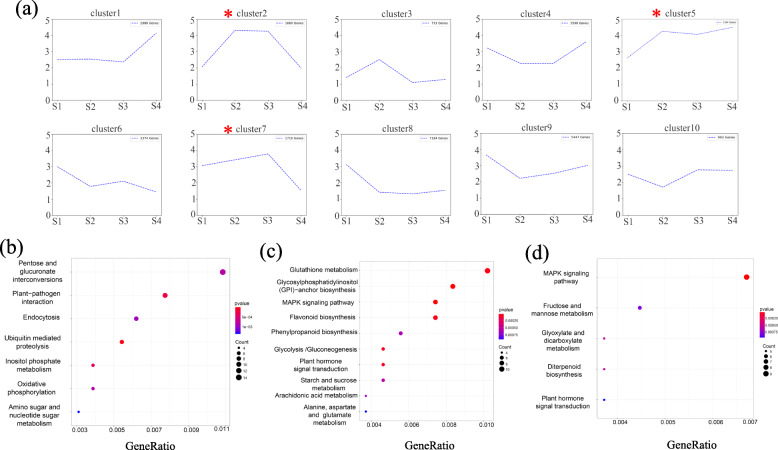
Transcript abundance of differentially expressed genes during flower development**. a** DEGs were classified into 10 clusters based on their expression profile. The cluster descriptions and DEG numbers for each cluster are designated. **b**-**c** KEGG enrichment of cluster 2 (**b**), cluster 5 (**c**) and cluster 7 (**d**). Clusters were classified using MeV (v4.9.0). Functional interpretation was further completed by KEGG (http://www.genome.jp/kegg/pathway.html)

To further investigate key genes that might regulate the physiological process of flower development and respond to pollinator visitation, we analyzed genes that were differentially expressed between all four stages and identified a total of 28,983 DEGs (Dataset 2). Then according to their expression pattern, they were classified into 10 clusters that can be generally divided into four types: upregulated (clusters 1 and 5), downregulated (clusters 6 and 8), up-then downregulated (clusters 2, 3 and 7) and down-then upregulated (clusters 4, 9 and 10) (Fig. 5a). Considering that flowers of *N. tangutorum* bloom and secrete nectar during stages 2 and 3, we focused on analyzing the genes in clusters 2, 5 and 7 which may facilitate us to explore targeted genes or pathways. Cluster 2 consisted of genes which were strongly expressed at stages 2 and 3. KEGG enrichment showed that ‘Pentose and glucuronate interconversions’ and ‘Plant-pathogen interaction’ were enriched in this cluster (Fig. 5b). For cluster 5, genes were highly expressed during stages 2–4, and the main functions of these genes were ‘Glutathione metabolism’, ‘Glycosylphosphatidylinositol (GPI)-anchor biosynthesis’, ‘MAPK signaling pathway’, ‘Flavonoid biosynthesis’, ‘Glycolysis/Gluconeogenesis’, ‘Plant hormone signal transduction’ and ‘Starch and sucrose metabolism’ (Fig. 5c). For cluster 7, genes were highly expressed at stage 3 and then quickly declined. The functions of these genes were ‘MAPK signaling pathway’, ‘Fructose and mannose metabolism’, ‘Glyoxylate and dicarboxylate metabolism’, ‘Diterpenoid biosynthesis’ and ‘Plant hormone signal transduction’ (Fig. 5d).

In addition, we identified a large number of DEGs and enzymes involved in flower and nectary development (*Agamous-like* (*AGL*) family *9*, *AGL11*, *AGL18*, *AGL27*, *AGL30*, *CRC*, *AP1*, *AP2*), starch and sucrose metabolism (beta-fructofuranosidase (*β-FFase*), GDSL esterase/lipase (*GELP*), glycosyltransferase (*GTs*), fructokinase (*FRK*), glucosidase (*GLU*), *CWINV4*, *SWEET2*, *SWEET5*, *SWEET6*, *SWEET7*, *SWEET9*, *SWEET14*, *SWEET15*), flavonoid biosynthesis (*FLS*), plant hormone signal transduction (*IAAs*, *SAUR*, BR-signaling kinases (*BSKs*), DELLA, *GID1*, jasmonate ZIM domain-containing protein (*JAZ*)) and plant-pathogen interaction (*RPS2*, *WRKY*) (Fig. S2). From the above results, we found that these genes were mainly enriched for sugar synthesis, interconversion and metabolism and antimicrobial activity.

**Fig. 6:**
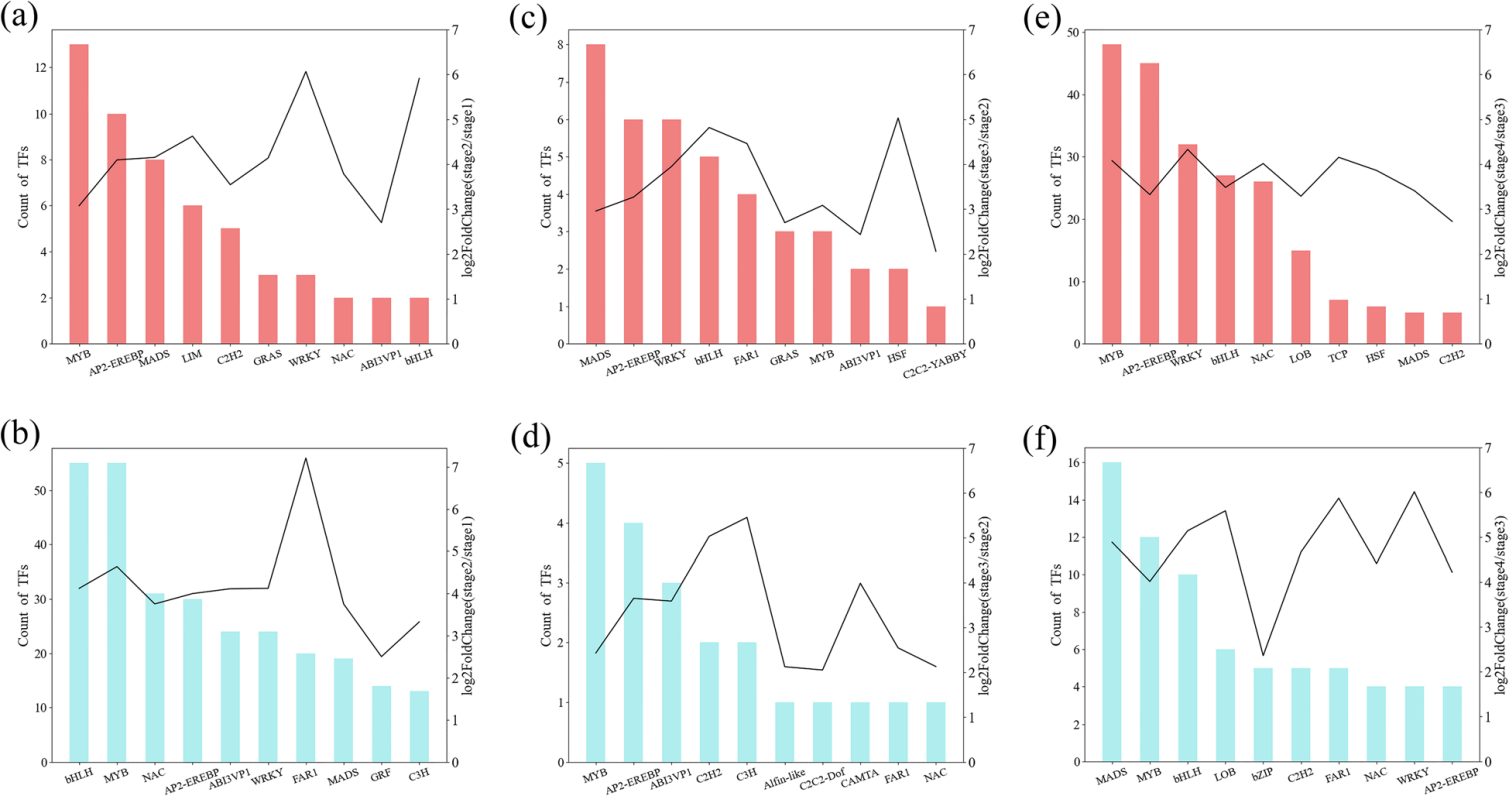
Transcription factor family prediction of differentially expressed genes. Histograms showing the number of DEGs belonging to a certain TF family at each flower developmental stage comparison (left y-axis, red bars for upregulated TFs, blue for downregulated), combined with their expression level fold change (right y-axis). **a**, **c**, **e** Up-regulated count of TFs between stages 1 and 2 (**a**), stages 2 and 3 (**b**) and stages 3 and 4 (**e**). **b**, **d**, **f** Down-regulated count of TFs between stages 1 and 2 (**b**), stages 2 and 3 (**d**) and stages 3 and 4 (**f**). Transcription factors (TF) were predicted using hmmsearch (https://www.ebi.ac.uk/Tools/hmmer/)

Flower development is usually regulated by various transcription factors (TFs); therefore, we predicted the DEGs that encode TFs and classified them according to their TF family (Fig. 6, Table S2). In our comparison of stages 1 to 2, the differentially expressed TFs mainly included genes from the *bHLH*, *MYB*, *NAC*, *AP2-EREBP*, *WRKY* and *MADS* families (Fig. 6a-b), with most of them being down-regulated in stage 2. Between stages 2 and 3, we could not detect significant differences in TF expression, and the number of differentially expressed TFs was very low (Fig. 6c-d). They were also involved in flower development, metabolic pathways and stress tolerance. Between stages 3 and 4, more TFs were up-regulated, mainly including *MYB*, *AP2-EREBP*, *WRKY*, *bHLH* and *NAC* (Fig. 6e). Down-regulated TF families were mainly *MADS*, *MYB* and *bHLH* (Fig. 6f).

### Identification and functional annotation of *Nitraria tangutorum* nectar proteins

From the previous transcriptome data, we detected the expression of a number of nectar synthesis and secretion and plant-pathogen interaction genes (Fig. S2). All these genes are related to nectar and its potential functions. We then used Coomassie Brilliant Blue R-250 staining to visualize nectar proteins within a Sodium dodecyl sulfate-Polyacrylamide gel electrophoresis (SDS-PAGE) gel. Protein sizes ranged from around 7 to 67 kDa, among two duplicated lanes we could distinguish nine distinct bands (Fig. S3). Then, we isolated the nine separate protein bands from the gel matrix and examined their contents via Liquid chromatography-tandem mass spectrometry (LC-MS/MS). We successfully obtained basic information (protein name, Protein accession, Protein molecular weight, Hit peptides) on the detected peptides and successfully completed the annotation of 41 proteins by searching the SWISS-PROT database (Table S3).

**Fig. 7:**
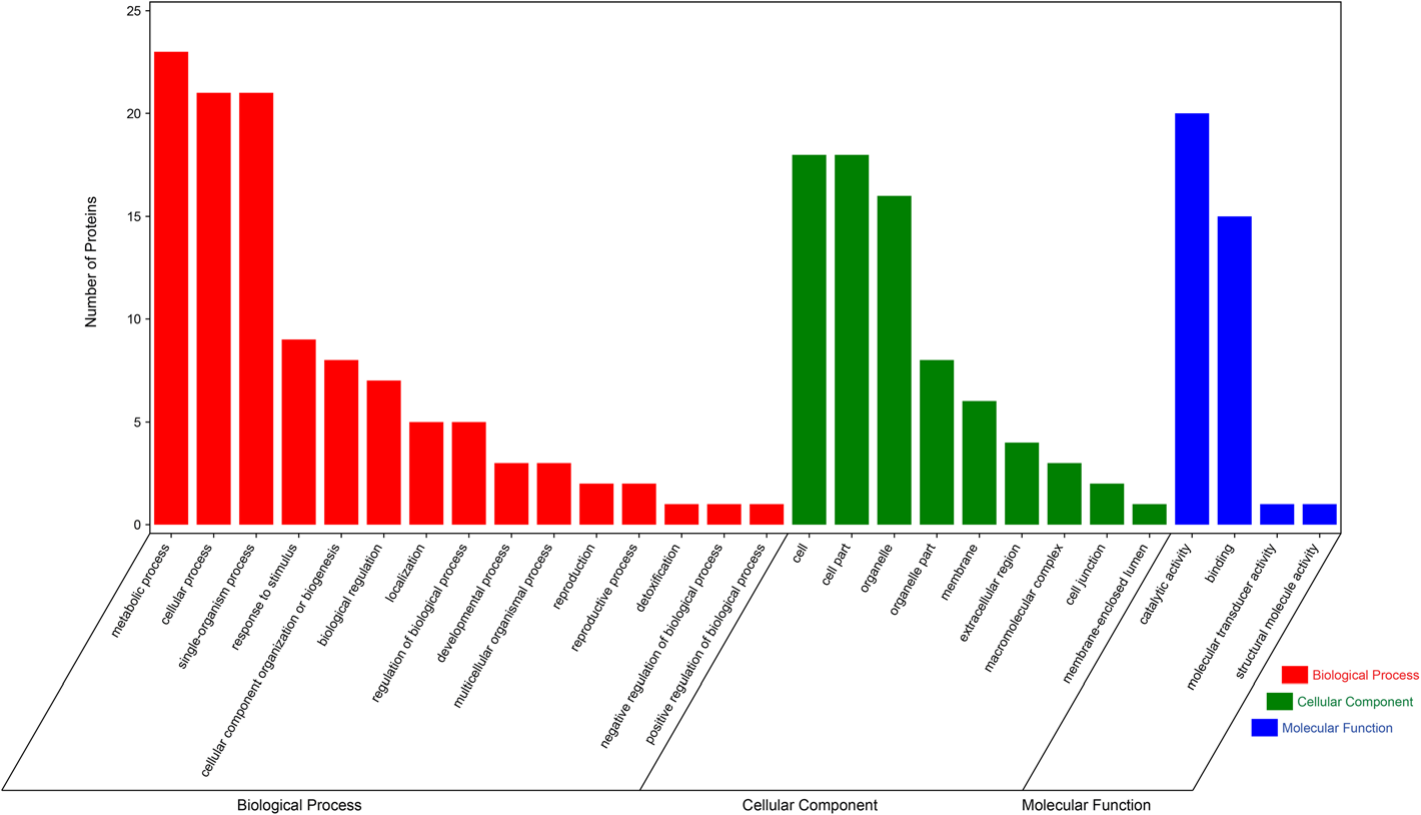
Functional classification of proteins identified in *Nitraria tangutorum* floral nectar. Histogram displaying the number of proteins identified in *Nitraria tangutorum* nectar represented by a GO term out of one the three main GO term categories: BP (red), CC (green) and MF (blue). Numbers on the Y-axes indicate protein numbers

Blast2GO assignments were used to annotate the nectar proteins with Gene Ontology (GO) terms, after which the identified proteins were categorized according to the three main GO categories (Fig. 7): biological process (BP), cellular component (CC) and molecular function (MF). For the BP category, most proteins were predicted to function in metabolic, cellular and single-organism processes, as well as response to stimulus. Concerning the CC category, we found most proteins being predicted to localize to the cell and organelle, followed by membranes, extracellular region, macromolecular complexes, cell junction and the membrane-enclosed lumen. Finally, within the MF category, most proteins likely possess catalytic and binding activity. In summary, the most highly represented GO categories among the identified proteins were metabolic, cellular, and single-organism processes, as well as catalytic activity (Fig. 7).

Furthermore, according to the position of the bands, the molecular weight of the proteins, obtained peptides and proteins reported in other plants, we selected seven interesting proteins (Table S3): SOD, MDAR, POD, REF, FBP2, nsLTPs and L-ascorbate oxidase (AO). It is worth mentioning that AO was first found in plant nectar. Taken together, these studies indicate that the nectar proteins of *N. tangutorum* may primarily have both an antibacterial and active oxygen scavenging effect.

### Validation of RNA-seq data and LC-MS/MS data by real time qPCR

**Fig. 8:**
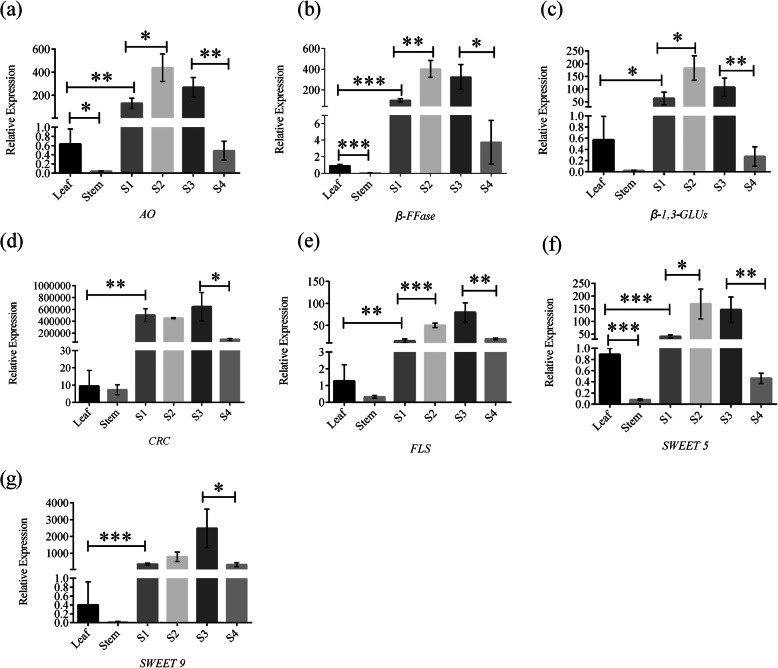
7 randomly selected DEGs were verified by RT-qPCR during flower development. **a** *L-ascorbate oxidase* (*AO*), **b** *beta-fructofuranosidase* (*β-FFase*), **c** *glucan endo-1,3-beta-glucosidase* (*β-1,3-GLUs*), **d** *CRABS CLAW* (*CRC*), **e** *flavonol synthase* (*FLS*), **f** *sugars will eventually be exported transporters 5* (*SWEET5*), **g** *sugars will eventually be exported transporters 9* (*SWEET9*). S1-S4 correspond to flower developmental stages in Fig. 1. Data represent means ± SD from three technical replicates. All the graphs were drawn using GraphPad Prism software (version 6.0). Statistical analysis of the data, shown as means ± SE, was conducted using one-way analysis of variance (ANOVA) and *t*-tests, taking *p < 0.05, **p < 0.01 and ***p < 0.001 as levels of significance

With the aim of validating the accuracy of our RNA-seq and LC-MS/MS data, we randomly determined the expression levels of several genes by qRT-PCR. *AO*’s expression was discoverable in the leaf and flowers, and was highest during stages 1–3 (Fig. 8a). For *β-FFase* and *β-1,3-GLUs*, their expression was highest at stage 2, and decreased at stages 3–4 (Figs. 8b and c). Furthermore, we found the expression level of *CRC* to be very high in flowers, while it was almost absent from the leaf and stem (Fig. 8d). Similarly, *FLS* was highly expressed during flower development compared to other tissues (Fig. 8e). Finally, the sugar transporters (*SWEET5* and *SWEET9*) were highly expressed in flower tissue as flower development progressed, yet decreased again strongly in expression towards the end of the flower’s life (Figs. 8f-g). A good correlation was observed between our qRT-PCR data and RNA-seq or LC-MS/MS data, which confirmed their high reliability.

### Key genes or enzymes involved in the physiological process of nectary development, nectar secretion and antimicrobial activity in *Nitraria tangutorum*

**Fig. 9:**
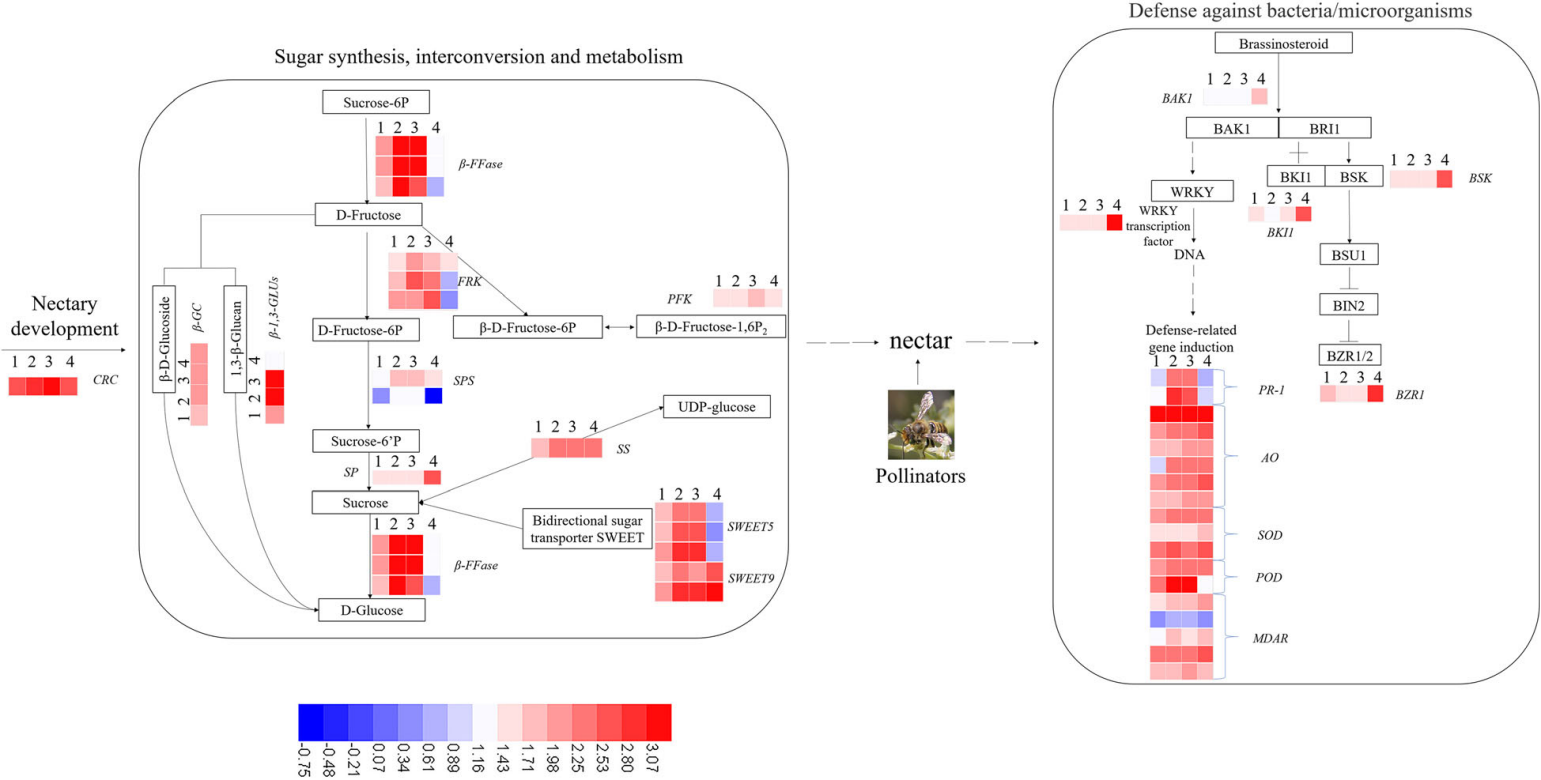
Physiological process of nectary development, nectar secretion and antimicrobial activity in *Nitraria tangutorum*. Relative expression (log_10_(FPKM)) profiles (blue-white-red) of genes. The figure was adapted from KEGG (http://www.kegg.jp/). The box represents the substance or gene that plays a role at each step. Sugar synthesis, interconversion and metabolism: comprising starch and sucrose metabolism; amino sugar and nucleotide sugar metabolism; fructose and mannose metabolism and glycolysis/gluconeogenesis. Antimicrobial activity and scavenging of active oxygen: comprising glutathione metabolism; plant hormone signal transduction and plant-pathogen interaction

Based on transcriptome and proteome analysis, we found that there were mainly two cascade networks composed of many pathways regulating the breeding system of *N. tangutorum* (Fig. 9). One of them is sugar synthesis, interconversion and metabolism, comprising starch and sucrose metabolism, amino sugar and nucleotide sugar metabolism, fructose and mannose metabolism and glycolysis/gluconeogenesis. The other is antimicrobial activity and scavenging of active oxygen, comprising glutathione metabolism, plant hormone signal transduction and plant-pathogen interaction. Primarily, the gene *CRC* which regulates nectary development was highly expressed across all four stages. When the nectary matures, it secretes nectar. Subsequently, sucrose synthase (*SS*) and sucrose phosphate synthase (*SPS*) synthesize sucrose, which is then hydrolyzed by beta-fructofuranosidase (*β-FFase*) to produce D-Fructose and D-Glucose. Further metabolism of fructose requires phosphorylation by fructokinase (*FRK*), which produces D-Fructose-6P and β-D-Fructose-6P. D-Fructose can also be converted to D-Glucose by β-D-Glucoside (*β-GC*) and 1,3-β-Glucan (*β-1,3-GLUs*). β-D-Fructose-6P and β-D-Fructose-1,6P_2_ can be converted into one another. D-Fructose-6P can be used for synthesis of Sucrose-6’P, which is catalyzed by sucrose-phosphate synthase (*SPS*). We further identified the bidirectional sugar transporters *SWEET 5* and *SWEET 9*. Most of these enzymes are highly expressed during stages 2 and 3.

As the nectar is secreted, it attracts pollinator’s visitation (Fig. 2). Insects may carry along bacteria, which activate the plant’s defense-related genes. It was found that the genes related to the brassinosteroid pathway (*BAK1*, *BKI1*, *BSK*, *BZR1*) had the highest expression level at stage 4, possibly due to it being a critical period for fruit formation. The *WRKY* TF had the highest expression level during stage 4 as well. Other defense-related genes, such as *PR-1*, *AO*, *SOD*, *POD* and *MDAR*, which we also detected in the nectar’s proteome, were highly expressed throughout the flower’s developmental stages 2 and 3. These results describe the physiological process of flower development in *N. tangutorum* at the gene expression level and showed the relevance of this study.

## Discussion

### The pollination ecology of *Nitraria tangutorum*

As a plant surviving from the Tertiary period, *Nitraria tangutorum* has been able to thrive up until the present day, making it of great significance to study its breeding behavior. *Nitraria tangutorum* has a large number of characteristics that help its sexual reproduction, such as flowers that are numerous but small, reducing flower wind resistance and preventing them from being blown off easily. The corolla is white, allowing for temperature adjustments inside the flower, which ensures pollen development, while also attracting insect pollination leading to successful fertilization. Previous studies reported that *N. tangutorum* has both hybrid affinity and self-compatibility, but there are no detailed reports on specific pollinators [29].

In this study, we found that the pollinators of *N. tangutorum* mainly include Hymenoptera, Diptera and Coleoptera (Fig. 2). It is likely that due to the small size and low volume of exposed nectar, *N. tangutorum* flowers mainly attract small insects. Furthermore, these insect species are found on most plants that grow in Inner Mongolia [30]. Their visiting behavior can promote the cross-pollination of *N. tangutorum*, which confirmed that it is an insect-pollinated plant.

### Gene regulation during flower development

Flower development involves many physiological processes that are regulated by a large number of genes. In this study, we found that the gradual opening of flowers, the synthesis of starch and the secretion of nectar that occurs during the transfer of stage 1 to 2 of *N. tangutorum* flower development, is associated with the differential expression of genes related to: ribosome biogenesis, indole alkaloid biosynthesis, ascorbate and aldarate metabolism, starch and sucrose metabolism and flavonoid biosynthesis. Genes related to metabolism and the synthesis and conversion of carbohydrates were differentially expressed between stages 2 and 3, which may be due to the plant’s large amount nectar secretion and its response to pollinator visits at this stage. Between stages 3 and 4, DEGs were mainly related to metabolic pathways, antimicrobial and resistance pathways, possibly due to the flowers having already fallen off and the normal development of fruits being more important. These results are similar to those that were found for the development of flowers from other plants [31, 32].

In addition, TF families widely occur in plants and are mainly involved in controlling plant growth, flower development, stress regulation, organ development and flavonoid biosynthesis, such as *bHLH*, *MYB*, *NAC*, *AP2-EREBP*, *MADS* and *WRKY* TF families [33]. Combining these findings with pathways we found to be enriched, it’s possible that at this stage, flower development is regulated by *MADS*, while *MYB* and *NAC* regulated flavonoid biosynthesis. *bHLH*, *MYB*, *WRKY* and *AP2-EREBP* are involved in antimicrobial and pathogen resistance pathways.

### The nectary of *Nitraria tangutorum* is composed of secretory trichomes

In this study, the nectary of *N. tangutorum*, a perianth nectary that is composed of secretory trichomes, was evaluated in detail (Fig. 3). Perianth nectaries are not that frequently found in the plant kingdom, the most common nectary being the stomatal nectary [34, 35]. The nectary is located at the petal base, making the secreted, exposed nectar more conducive to insect foraging. Furthermore, the secretory trichomes may prevent the evaporation of nectar. These results are consistent with previous research [25].

Nectaries occur in many different shapes and sizes across the plant kingdom [36, 37]. Their position within the flower and structure various greatly and many genes are known to regulate their development. We found five genes of the *AGL* family, *AP2* and *CRC* to be expressed dynamically during *N. tangutorum* nectary development (Fig. S2).

### The synthesis and secretion of *Nitraria tangutorum* nectar and its functions

Our results found some enzymes and genes associated with the synthesis and secretion of *N. tangutorum* nectar, including *β-FFase*, *GELP*, *GTs*, *FRK*, *BGL*, *CWINV4*, *SWEET2*, *SWEET5*, *SWEET6*, *SWEET7*, *SWEET9*, *SWEET14* and *SWEET15* (Fig. S2). To date, no studies have investigated *N. tangutorum* nectar proteins. Here, we found the nectar protein bands to be numerous and complex, forming a total of nine distinct bands on a 1D protein gel. We identified a total of seven interesting proteins in the nectar of *N. tangutorum*, six of which have been previously found in the nectar of other plants [22, 24, 38–40] (SOD, MDAR, POD, REF, FBP2, nsLTPs), and we identified AO in floral nectar for the first time. A previous study has shown that nectar proteins mainly have two functions: post-secretory hydrolysis of nectar sugars for pollinators and defense against microorganisms [40]. We found that the nectar proteins of *N. tangutorum* may have both an antibacterial effect, as well as an oxygen scavenging effect. It may have been possible for *N. tangutorum* to respond to a specific growth environment by secreting a wider variety of nectar proteins. If nectar does not provide a strong antibacterial defense, microorganisms will grow uninhibited and directly change the nectar composition, which thereby loses its attractiveness to pollinators and lowers plant reproduction. These findings provide important information on *N. tangutorum* nectar composition, and show that proteins known to occur in the nectar of other plants may also have a function in desert plants.

## Conclusions

In summary, this study revealed that *N. tangutorum* is an insect-pollinated plant, and that pollinators of *N. tangutorum* mainly include Hymenoptera, Diptera and Coleoptera. In addition, its nectary is composed of secretive epidermal cells that specialized to secretive trichomes and secrete nectar. The transcriptome data of successive flower developmental stages were compared, and DEGs controlling flower and nectary development, nectar biosynthesis and secretion, flavonoid biosynthesis, plant hormone signal transduction and plant-pathogen interaction were identified. Furthermore, we identified several proteins present in *N. tangutorum* nectar and found that they may have both an antibacterial and oxygen scavenging effect. The results will benefit research into the breeding behavior of arid-desert plants in general and provide insight into how *N. tangutorum* deal with its harsh environment specifically.

## Materials and methods

### The *Nitraria tangutorum* habitats and sample collection

*Nitraria tangutorum* is a shrub with a large number of densely packed branches, each plant having tens of thousands of flowers. When the branches are covered with sand, adventitious roots easily sprout downward and adventitious buds sprout upward. *Nitraria tangutorum*’s natural habitat is in Dengkou, within the Inner Mongolia Municipality (106°09′-107°10′ E, 40°09′-40°57′N). This region is characterized by long hours of sunshine, large temperature differences between day and night, and heavy rainfall. The annual average temperature is 9.8 °C. The average precipitation in the most recent 10 years is 150.6 mm. The soil here is mainly sandy and the vegetation is dominated by xerophytic shrubs. *Nitraria tangutorum* is one of the dominant species in this area, with some of the other native species being *Ammopiptanthus mongolicus* Maxim., *Artemisia ordosica* Krasch. and *Artemisia sphaerocephala* Krasch.

The author Jingbo Zhang was responsible for the formal identification of the samples. However, to our knowledge, there is no herbarium to deposit the voucher specimen of this specific material. All materials were obtained with permission.

### Floral nectar collection and floral visitor species/ behavioral observations

To prevent nectar from being contaminated by pollinators or other contaminants, we surrounded flowers with transparent bags before blooming. Raw *N. tangutorum* nectar was collected from blooming flowers at 5:00–7:00 am. To avoid the evaporation of nectar caused by high temperature, we gently collected droplets of nectar using a 10 μl sterile plastic micropipette. Nectar was placed in a 0.5 ml centrifuge tube, which was then quickly stored in liquid nitrogen. We collected nectar from a very large number of flowers due to their small size and the low quantity of nectar per flower.

During the blooming period (from May 20nd to June 10th), three plants with roughly the same growth habit were randomly selected, and each plant was observed by one person at a fixed point for 1 week. When observed flower-visiting behavior lasted for more than 10s, floral visitors were photographed and recorded using an infrared camera. According to the time of activity of Hymenoptera, Diptera, Coleoptera and other insects, we selected sunny days from 7:00 to 17:00 for observation and recording.

### Nectary morphology study using scanning electron microscopy

For SEM investigation, fresh flower samples (approximately 10–20 flowers per stage) from three different plants were fixed in 4% glutaraldehyde fixative, dehydrated with a graded alcohol series, dried to the CO_2_ critical point and plated with gold. Photographs were taken by a Quanta 200 environmental scanning electron microscope [40].

### Transcriptome sequencing and gene annotation

Whole *Nitraria tangutorum* flowers were collected based on four developmental stages, collecting three replicates from three plants (approximately 10–15 flowers per replicate) for each developmental stage, after which they were immediately frozen in liquid nitrogen and stored at − 80 °C until use. These samples were then sent for transcriptome sequencing at BGI (Wuhan, China). For the purification of total RNA from whole flowers, ethanol precipitation and CTAB-PBIOZOL reagent were used. For RNA concentration and quality checks, a NANO DROP was used. Total RNA was used for sequencing library construction, with the total RNA amount ≥ 1 μg. After library quality inspection, Illumina sequencing was performed. SOAPnuke (v1.5.2) was used to filter the adapter, low quality and high N proportion reads. HISAT2 (v2.0.4) was used to construct the reference genome (unpublished data) index for read mapping. Next, we quantified the transcripts using RSEM (v1.2.12). In addition, sample repeatability, gene expression levels and gene expression distribution for each sample were estimated or measured. Blast2GO (http://www.biobam.com/blast2go-command-line-tools/) was used for the annotation of sequencing data. Gene differential expression analysis was performed using NOIseq (https://bioinfo.cipf.es/noiseq/doku.php?id=downloads). The significance threshold was set at |Log_2_FoldChange| ≥ 1 and Probability ≥0.75. Clusters were classified using MeV (v4.9.0). Functional interpretation was further completed by KEGG (http://www.genome.jp/kegg/pathway.html). Heat maps were constructed using TBtools (v1.0) software. For transcription factor (TF) prediction of DEGs, we first identified the DEGs ORFs, then aligned these ORFs to TF domains using hmmsearch (https://www.ebi.ac.uk/Tools/hmmer/).

### 1-D gel electrophoresis and LC-MS/MS analysis

Owing to the small size of *Nitraria tangutorum* flowers, it was hard to obtain a high volume of nectar. Amicon Ultra 3 K centrifugal filter devices (Millipore, USA) were used to purify and concentrate nectar, then protein content was determined according to previously published methods [41]. Sample buffer was used to boil the concentrated nectar (10 μl, ~ 20 μg total protein) for 5 min, after which it was analyzed by SDS-PAGE 1-D gel electrophoresis. For target protein identification, gel sections having proteins were manually extracted from 1-D gels and sent to BIOMS (Beijing, China) for LC-MS/MS analysis. Zhou [40] method was used to process the data and protein annotation.

### Real time quantitative PCR analysis

Seven DEGs from our RNA-seq analysis were selected for analysis of their expression using RT-qPCR. Leaf, stem and flower tissue of four stages were collected to sample expression. RNA was extracted, denatured, and first-strand cDNA was synthesized using the HiScript II 1st Strand cDNA Synthesis Kit (Vazyme, China). RT-qPCR was performed using a LightCycler 480 II Real-Time PCR System (Rioche, USA). The SYBR method was used and the gene expression level was calculated with the 2^−ΔΔCT^ method [40]. Primer sequences were designed using Oligo 7 and are listed in Table S2. All samples were examined in (technical) triplicate, using three biological replicates. *Actin* was used as a reference gene.

### Statistical analysis

All data were analyzed using GraphPad Prism software (version 6.0). Statistical analysis of the data, shown as means ± SE, was conducted using one-way analysis of variance (ANOVA) and *t*-tests, taking *p < 0.05, **p < 0.01 and ***p < 0.001 as levels of significance.

## Supplementary Information

**Additional file 1: Fig. S1**. RNA-seq data validation. (a) Pearson correlations between different samples (4 developmental stages (S1-S4) with 3 biological replicates (rep1-rep3)), (b) Boxplot showing the gene expression level distribution for each sample, (c) Hierarchical clustering of DEGs between comparisons of different developmental stages

**Additional file 2: Fig. S2**. Heatmap of the expression levels (log2(FPKM)) of DEGs related to flower and nectary development, starch and sucrose metabolism, flavonoid biosynthesis, plant hormone signal transduction and plant-pathogen interaction. Heat maps were constructed using TBtools (v1.0) software

**Additional file 3: Fig. S3**. Gel electrophoresis of *N. tangutorum* proteins. M: maker, 1, 2: nectar protein replicates

**Additional file 4: Dataset 1**. Expression level and function annotation of all identified genes. The gene expression level at each stage is the average of three replicates. Blast2GO (http://www.biobam.com/blast2go-command-line-tools/) was used for the annotation of sequencing data

**Additional file 5: Dataset 2**. Differentially expressed genes during flower development. The gene expression level at each stage is the average of three replicates

**Additional file 6: **S**upplemental Table 1**. Sample description and RNA-seq reads number

**Additional file 7: Supplemental Table 2**. Transcription factors prediction in different flower development stage comparative groups. Transcription factors (TF) were predicted using hmmsearch (https://www.ebi.ac.uk/Tools/hmmer/)

**Additional file 8: Supplemental Table 3**. Floral nectar proteins of *N. tangutorum* annotated by LC-MS/MS after 1-D gel electrophoresis

**Additional file 9: Supplemental Table 4**. Primers used in qRT-PCR

## Data Availability

All data generated or analyzed during this study are included in files and NCBI SRA database (Accession number: PRJNA686177, https://submit.ncbi.nlm.nih.gov/subs/sra/SUB8746254/overview), and are available by contacting with the corresponding author (chenjh@njfu.edu.cn) on reasonable request.

## Abbreviations

DEG: Differentially Expressed Genes
TF: Transcription Factor
SEM: Scanning Electron Microscopy
KEGG: Kyoto Encyclopedia of Genes and Genomes
RT-qPCR: Real time quantitative PCR
ORF: Open Reading Frame
GO: Gene Ontology
FPKM: Fragments Per Kilobase of exon per Million reads mapped
SDS-PAGE: Sodium dodecyl sulfate -Polyacrylamide gel electrophoresis
LC-MS/MS: Liquid chromatography-tandem mass spectrometry
