# Supplementary figures and images for: Integrative analysis of transcriptome and proteome revealed nectary and nectar traits in the plant-pollinator interaction of Nitraria tangutorum Bobrov
Source: BMC Plant Biol. 2021 May 22;21:230. doi: 10.1186/s12870-021-03002-9 (PMC8140516; doi:10.1186/s12870-021-03002-9)

(a)

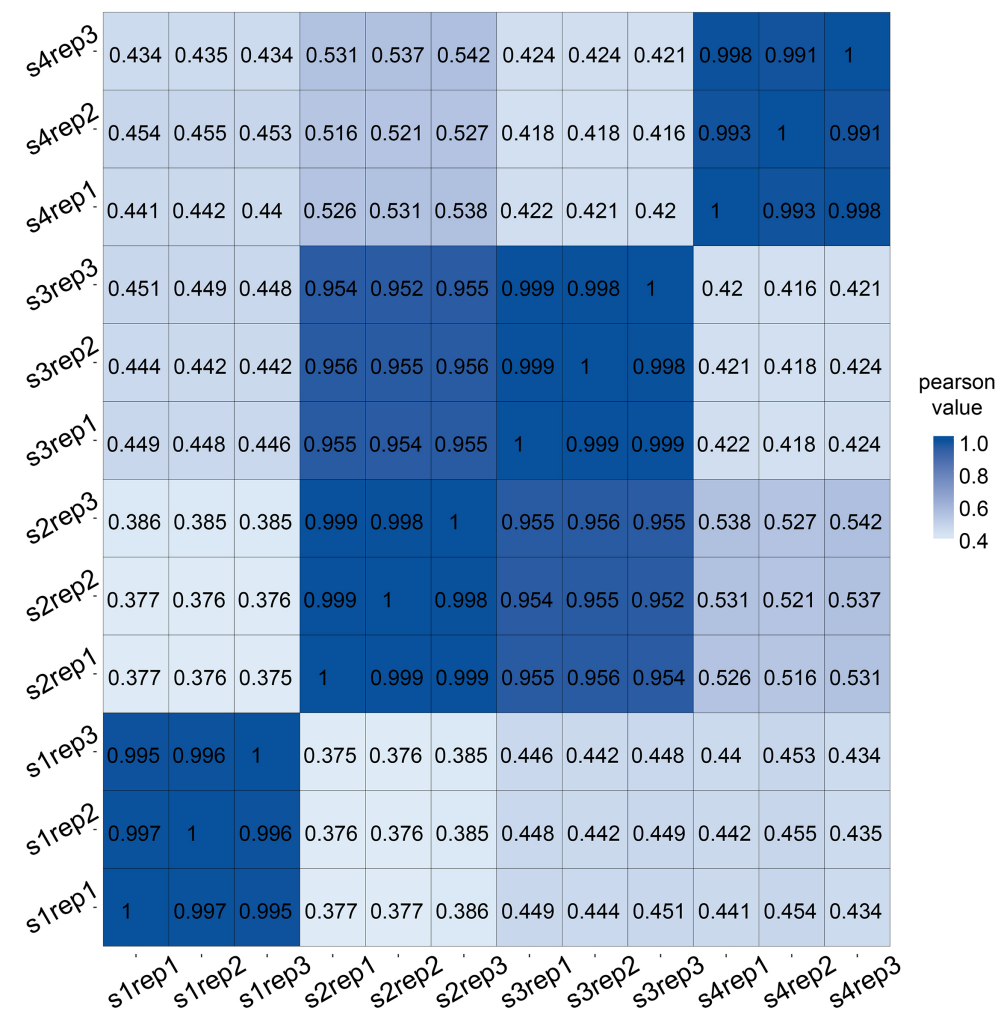

(b)

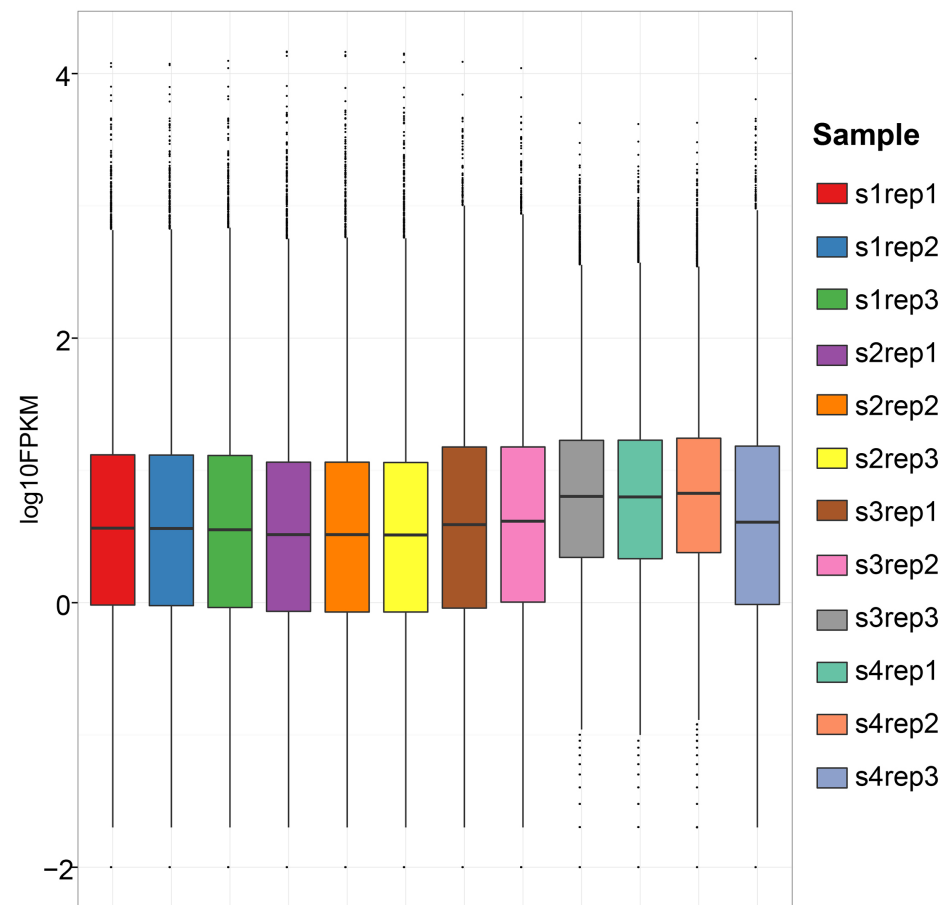

(c)

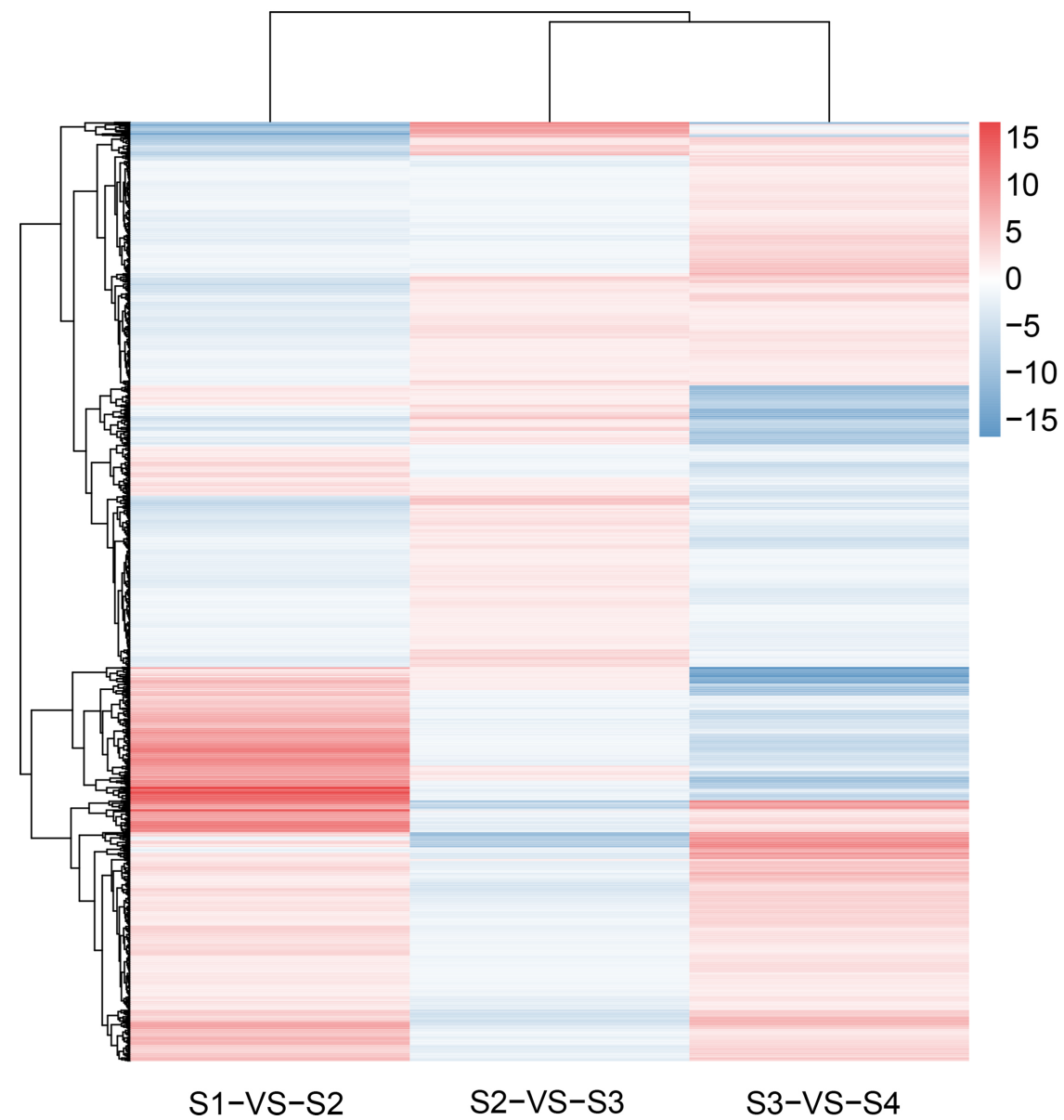

Supplement: Supplementary file 1 — Additional file 1: Fig. S1. RNA-seq data validation. (a) Pearson correlations between different samples (4 developmental stages (S1-S4) with 3 biological replicates (rep1-rep3)), (b) Boxplot showing the gene expression level distribution for each sample, (c) Hierarchical clustering of DEGs between comparisons of different developmental stages [file 12870_2021_3002_MOESM1_ESM.pdf]

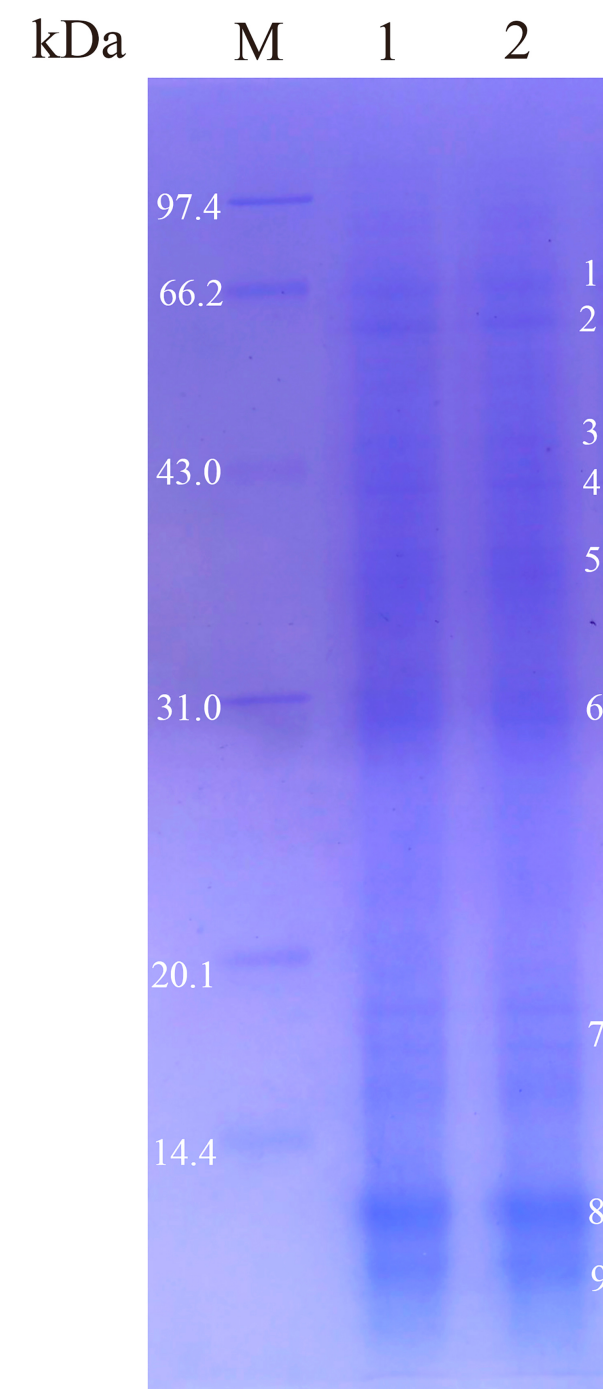

Supplement: Supplementary file 3 — Additional file 3: Fig. S3. Gel electrophoresis of N. tangutorum proteins. M: maker, 1, 2: nectar protein replicates [file 12870_2021_3002_MOESM3_ESM.pdf]
