# Supplementary material for: Integrative analysis of transcriptome and proteome revealed nectary and nectar traits in the plant-pollinator interaction of Nitraria tangutorum Bobrov
Source: BMC Plant Biol. 2021 May 22;21:230. doi: 10.1186/s12870-021-03002-9 (PMC8140516; doi:10.1186/s12870-021-03002-9)
Supplement: Supplementary file 2 — Additional file 2: Fig. S2. Heatmap of the expression levels (log2(FPKM)) of DEGs related to flower and nectary development, starch and sucrose metabolism, flavonoid biosynthesis, plant hormone signal transduction and plant-pathogen interaction. Heat maps were constructed using TBtools (v1.0) software [file 12870_2021_3002_MOESM2_ESM.pdf]

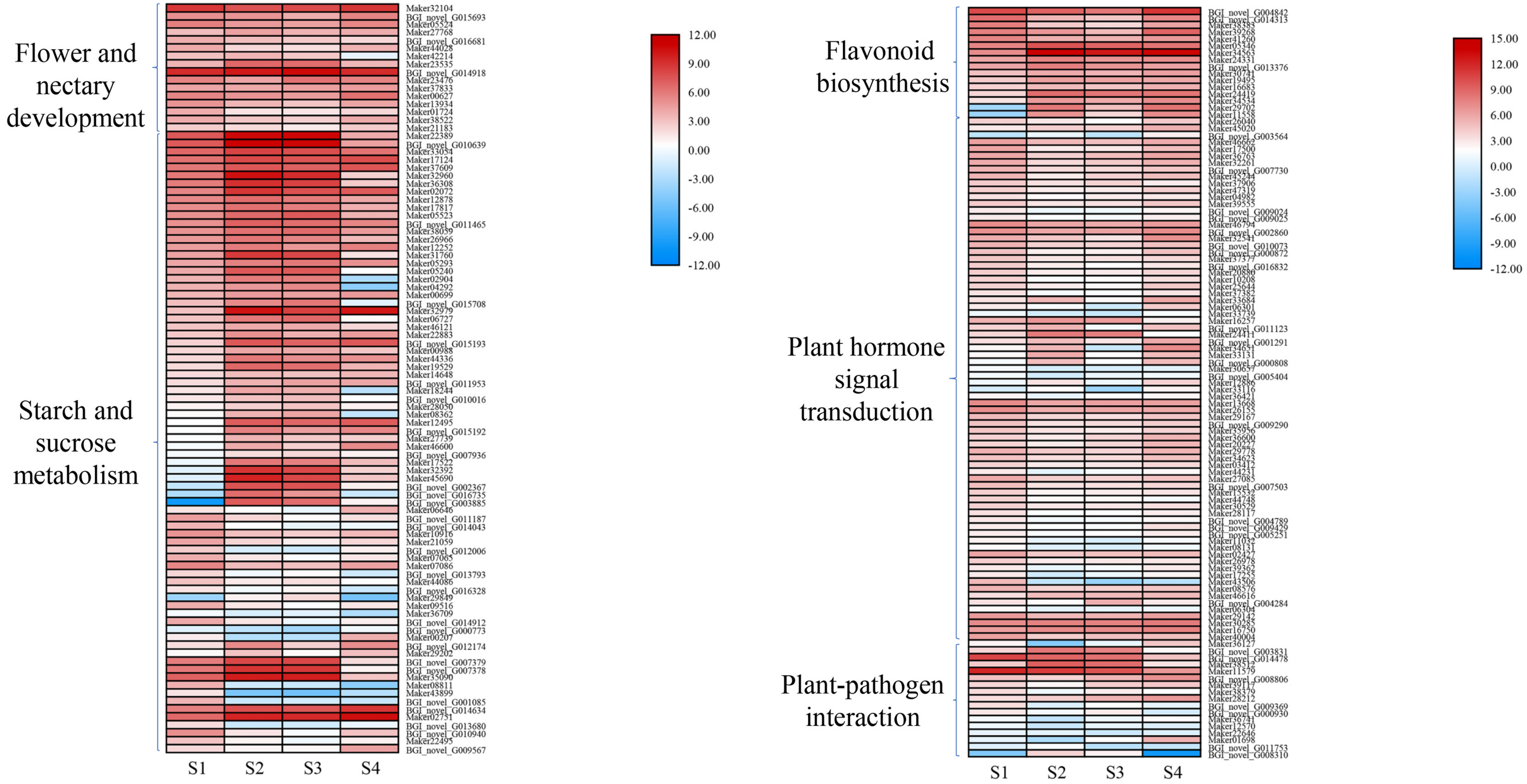

Flower and nectary development
Starch and sucrose metabolism
S1
S2
S3
S4
12.00
9.00
6.00
3.00
0.00
-3.00
-6.00
-9.00
-12.00
Flavonoid biosynthesis
Plant hormone signal transduction
Plant-pathogen interaction
S1
S2
S3
S4
15.00
12.00
9.00
6.00
3.00
0.00
-3.00
-6.00
-9.00
-12.00
